# The endocannabinoid anandamide has an anti-inflammatory effect on CCL2 expression in vascular smooth muscle cells

**DOI:** 10.1007/s00395-020-0793-3

**Published:** 2020-04-22

**Authors:** Beatrice Pflüger-Müller, James A. Oo, Jan Heering, Timothy Warwick, Ewgenij Proschak, Stefan Günther, Mario Looso, Flávia Rezende, Christian Fork, Gerd Geisslinger, Dominique Thomas, Robert Gurke, Dieter Steinhilber, Marcel Schulz, Matthias S. Leisegang, Ralf P. Brandes

**Affiliations:** 1 Fachbereich Medizin, Institute for Cardiovascular Physiology, Goethe University, Theodor-Stern-Kai 7, 60590 Frankfurt, Germany; 2 German Center for Cardiovascular Research (DZHK), Partner site Rhein Main, Frankfurt, Germany; 3 Max-Planck-Institute for Heart- and Lung Research (MPI-HLR), 61231 Bad Nauheim, Germany; 4 Branch for Translational Medicine and Pharmacology TMP, Fraunhofer Institute for Molecular Biology and Applied Ecology IME, 60438 Frankfurt, Germany; 5 Faculty of Medicine, Pharmazentrum Frankfurt/ZAFES, Institute of Clinical Pharmacology, Goethe-University, 60590 Frankfurt, Germany; 6 Institute of Pharmaceutical Chemistry, Goethe-University, 60438 Frankfurt, Germany; 7 Vascular Research Centre, Goethe-University, 60596 Frankfurt, Germany

**Keywords:** Anandamide, HDAC4, NCoR1, Inflammation, CCL2

## Abstract

Endocannabinoids are important lipid-signaling mediators. Both protective and deleterious effects of endocannabinoids in the cardiovascular system have been reported but the mechanistic basis for these contradicting observations is unclear. We set out to identify anti-inflammatory mechanisms of endocannabinoids in the murine aorta and in human vascular smooth muscle cells (hVSMC). In response to combined stimulation with cytokines, IL-1β and TNFα, the murine aorta released several endocannabinoids, with anandamide (AEA) levels being the most significantly increased. AEA pretreatment had profound effects on cytokine-induced gene expression in hVSMC and murine aorta. As revealed by RNA-Seq analysis, the induction of a subset of 21 inflammatory target genes, including the important cytokine CCL2 was blocked by AEA. This effect was not mediated through AEA-dependent interference of the AP-1 or NF-κB pathways but rather through an epigenetic mechanism. In the presence of AEA, ATAC-Seq analysis and chromatin-immunoprecipitations revealed that CCL2 induction was blocked due to increased levels of H3K27me3 and a decrease of H3K27ac leading to compacted chromatin structure in the CCL2 promoter. These effects were mediated by recruitment of HDAC4 and the nuclear corepressor NCoR1 to the CCL2 promoter. This study therefore establishes a novel anti-inflammatory mechanism for the endogenous endocannabinoid AEA in vascular smooth muscle cells. Furthermore, this work provides a link between endogenous endocannabinoid signaling and epigenetic regulation.

**Electronic supplementary material:**

The online version of this article (10.1007/s00395-020-0793-3) contains supplementary material, which is available to authorized users.

## Introduction

Endocannabinoids are unsaturated fatty acid derivatives that represent an important class of signaling lipid mediators best known for their effects in the central nervous system [18]. It is well appreciated that endocannabinoids also have numerous effects directly on the cardiovascular system [26, 27]: endocannabinoids mediate intercellular and bidirectional communication in the brain [23], alter the vasomotor response [34] and cellular gene expression. The effects of endocannabinoids are largely mediated by the G_i_-coupled receptors CB1 and CB2 [6, 22] and to some extent by nuclear receptors like PPARs, which have been shown to be activated by endocannabinoids [25]. As endocannabinoids are released and taken up by different cells, these receptors can mediate autocrine and paracrine effects [10]. Since many cells, including those of the vascular system, produce and degrade endocannabinoids, these compounds have become interesting targets for the treatment and prevention of cardiovascular disease. Numerous studies have looked into the vascular consequences of a genetic knockout or activation of different endocannabinoid receptors and producing and degrading enzymes, with focus on inflammatory responses and atherosclerosis [24, 30]. However, the results were complex in that no unifying picture emerged. This is, in part, a consequence of the fact that the receptors all have different functions and that usually more than one endocannabinoid is produced by a cell. Depending on the presence of degrading and producing enzymes, cells can simultaneously produce 2-arachidonylglycerol (2-AG), arachidonoyl-ethanolamine (AEA), also known as anandamide, palmitoylethanolamide (PEA) and oleoylethanolamine (OEA) and each of these endocannabinoids can activate multiple receptors and signaling cascades.

In the present study we set out to identify novel signaling mechanisms that are activated by endocannabinoids in vascular smooth muscle cells. We found that AEA has a unique anti-inflammatory function which is mediated by an interesting epigenetic mechanism.

## Materials and methods

### Reagents

The following reagents were used: Human recombinant Interleukin-1 (IL-1)-β (#200-01B, Peprotech), murine recombinant Interleukin-1 (IL-1)-β (#211-11B, Peprotech), human recombinant tumor necrosis factor (TNF)-α (#300-01A, Peprotech), murine recombinant tumor necrosis factor (TNF)-α (#315-01A, Peprotech) Lipopolysaccharide from E-coli (#L3024, Sigma), Collagen Type I Rat Tail (#354236, Corning Incorporated), *N*-arachidonoylethanolamine (Anandamide, AEA, Sigma-Aldrich, #A-0580-25MG), RGFP966 (#16917.00, Cayman Chemicals), MC1568 (27761-1, BPS Bioscience), Diclofenac sodium salt (#D6899, Sigma).

The used antibodies can be find in supplemental Table 4. For Western blot antibodies were diluted 1:1000. For immunofluorescence and PLA antibodies were diluted in standard way 1:500 except for HDAC4 1:1000. ChIP experiments were performed with standard 3 µg of antibody except for NCoR1 were we used 6 µg. Primers for RT-qPCR, used siRNA and primers for ChIP PCRs are listed in Supplemental Tables 1, 2 and 3.

### Cell stimulation

HAoSMC and freshly isolated murine aortic smooth muscle cells were exposed to media with l-glutamine and 1% FCS overnight or for at least 16 h. The next day cells were incubated in media with 1% FCS, l-glutamine and 10 µM diclofenac (Sigma Aldrich, #D-6899) for 1 h and treated with 100 nM AEA or EtOH as solvent for 2.5 h. Diclofenac was used to prevent cyclooxygenase-mediated breakdown of AEA. Subsequently, 10 ng/mL IL-1β, TNFα (10 ng/mL or LPS (10 µg/mL) was added for 1.5 h. For protein measurements 100 nM AEA (1 h) and 10 ng/mL IL-1β treatment (3 h) in the presence of 10 µM diclofenac was used. For immunofluorescence and PLA measurements 100 nM AEA 2.5 h and 1 h 10 ng/mL IL-1β treatment in the presence of 10 µM diclofenac was used. Murine aortic rings were cultured in media without supplements and with 0.1% BSA. Treatment was performed in the presence of 10 µM diclofenac and pre-treatment with 100 nM AEA overnight. The next day, murine IL-1β (10 ng/mL), murine TNFα (10 ng/mL) or LPS (10 µg/mL) was added and incubated for 1.5 h.

### Cell culture

Human aortic smooth muscle cells (HAoSMC) (#354-05a) were purchased from PELOBiotech (Planegg, Germany). The cells were cultured on collagen-coated dishes in PELOBiotech Smooth Muscle Cell Medium (#PB-MH-200-2190) with 8% heat-inactivated fetal calf serum (FCS), l-glutamine, penicillin (50 U/ml) and streptomycin (50 µg/ml) (#15140-122, Gibco lifetechnologies, Carlsbad, CA, USA), EGF, bFGF and insulin from singlequots (PELOBiotech, Planegg, Germany) in a humidified atmosphere of 5% CO_2_ at 37 °C. For experiments cells from passage 5 to 10 were used. For each experiment at least three to five different batches (each a different donor) of HAoSMC were used. After siRNA transfection cells were transferred to media without penicillin/streptomycin.

### Experimental animals

All experimental procedures were approved by the local governmental authorities and were performed in accordance with the animal protection guidelines. Mice were housed in a specified pathogen-free facility with 12 h light–dark cycle and free access to chow and water. For these studies 4–6 weeks old male mice of the C57BL/6J strain were used.

### Cell isolation of murine aortic smooth muscle cells and organ culture

Mice (four animals per preparation) were killed with isoflurane overdose, aortas were removed and adhering connective tissue removed. For organ culture, the aorta was cut into 1 mm rings. These were cultured for 16 h in endothelial basal medium (EBM) with 0.1% BSA, 10 µM diclofenac and treated as already described. For cell isolation, vessels were cut open, the endothelium was scraped of and the tissue was extensively washed. Subsequently, the aortae were minced in 50 µL sterile MCDB medium with 0.1% BSA into 1 mm small pieces and subjected to collagenase dissociation (380 U/mL) for 40–60 min at 37 °C with trituration every 10 min. The supernatant of this procedure was pelleted and cultured in SMC basal medium from PELOBiotech (#PB-MH-200-2190) with 8% FCS and insulin in fibronectin coated dishes.

### Monocyte migration: Boyden chamber

THP-1 cells were cultured in RPMI medium 1640 + GlutaMAX from Gibco supplemented with 8% fetal calf serum (FCS), 10 mM HEPES, 1 mM sodium pyruvate (sigma) and streptomycin (50 μg/mL) in a humidified atmosphere of 5% CO_2_ at 37 °C. THP-1 cell migration was studied in a modified Boyden chamber assay using a transwell chamber system (Fluoroblock, 8 µM pore size, BD Bioscience, Heidelberg, Germany). 2.5 × 10^4^ cells were seeded in the insert in RPMI medium with 0.1% BSA. Migration towards the stimulus or supernatant derived from HAoSMC conditioned media which was added to the lower chamber, was determined after 14 h. For conditioned media HAoSMC were treated with 100 nM AEA (2 h) and 10 ng/mL IL-1β (3 h). For further analysis supernatant was incubated with 50 ng/mL recombinant human CCL2 (#300-04, Peprotech) or 1:1000 diluted CCL2 antibody (#sc-52877, Santa Cruz).

### Chromatin immuno-precipitation (ChIP) in HAoSMC

HAoSMC were incubated in media without supplements and additional 1%FCS overnight. The next day 10 µM diclofenac was added and incubated for 1 h. Afterwards cells were treated with 100 nM AEA for 2 h and 1 h with 10 ng/mL IL-1β. Cells were crosslinked with formaldehyde (1%, 10 min). To stop crosslinking glycine (0.1 M, 5 min) was added. The cells were washed twice with cold DPBS (without calcium and magnesium), scraped and transferred to Falcon tubes and centrifuged for 5 min at 800 × g RT. Nuclear isolation was performed with truCHIP™ Chromatin Shearing Kit (Covaris, Woburn, USA) according to the manufacturers protocol. Afterwards, the lysates were sonified with a Bioruptor Plus (ten cycles, 30 s on, 90 s off; Diagenode, Seraing, Belgium) at 4 °C. Debris were removed by centrifugation. Chromatin content was measured by QuBit to adjust the chromatin concentration for each tube. The lysates were diluted 1:3 in dilution buffer (20 mmol/L Tris/HCl pH 7.4, 100 mmol/L NaCl, 2 mmol/L EDTA, 0.5% Triton X-100 and protease inhibitors). After pre-clearing with 25 μL DiaMag protein A-coated magnetic beads (Diagenode, Seraing, Belgium) for 1 h at 4 °C, samples were incubated overnight at 4 °C with 3 μg of antibody. 5% of the samples served as input. The antibody complexes were collected with 50 μL DiaMag protein A-coated magnetic beads (Diagenode, Seraing, Belgium) for 3 h at 4 °C, subsequently washed twice for 10 min with each of the wash buffers 1–3 (Wash Buffer 1: 20 mmol/L Tris/HCl pH 7.4, 150 mmol/L NaCl, 0.1% SDS, 2 mmol/L EDTA, 1% Triton X-100; Wash Buffer 2: 20 mmol/L Tris/HCl pH 7.4, 500 mmol/L NaCl, 2 mmol/L EDTA, 1% Triton X-100; Wash Buffer 3: 10 mmol/L Tris/HCl pH 7.4, 250 mmol/L lithium chloride, 1% Nonidet, 1% sodium deoxycholate, 1 mmol/L EDTA) and finally washed with TE-buffer pH 8.0. Elution of the beads was performed with elution buffer (0.1 M NaHCO3, 1% SDS) containing 1 × proteinase K (Diagenode, Seraing, Belgium) and shaking at 600 rpm for 1 h at 55 °C, 1 h at 62 °C and 10 min at 95 °C. Subsequently, the eluate was purified with the QiaQuick PCR purification kit (Qiagen, Hilden, Germany) and subjected to qPCR analysis (qPCR Primers: Supplemental Table 3, antibodies: Supplemental Table 4).

### Immunofluorescence staining in HAoSMC

HAoSMC were treated as already described for ChIP experiments. Cells were fixed by adding paraformaldehyde in PBS to a final concentration of 2% for 10 min. Cells were permeabilized with 0.05% Triton X-100 for 10 min and blocked for 30 min with 3% BSA. The NCoR1 antibody (#A301-145A) or HDAC4 (#A303-467A) was incubated overnight at 4 °C. The secondary antibody (incubation 1 h) and DAPI staining (incubation 10 min) were performed the next day after several washing steps with PBS and 0.3% Tween. Cells were stored in PBS with 0.02% sodium azide. Images were acquired with a Zeiss LSM800 laser scanning microscope.

### Proximity ligation assay

Human aortic smooth muscle cells were seeded onto 8-well ibidi slides. The next day, cells were treated for 2.5 h with 100 nM AEA and subsequently for 1 h with 10 ng/mL IL-1β. All experiments were done with preincubation of 10 µM diclofenac. Cells were fixed and permeabilized as already described for immunofluorescence and blocked with blocking buffer of the Duolink In Situ Detection Reagents Orange Kit (#DU92007, Sigma Aldrich). Incubation with primary antibodies against HDAC4 (rabbit, A303-467A, Bethyl) and NCoR1 (LSBio, #LS-C108878-100) was performed overnight at 4 °C. The next day after washing with PBS and 0.3% Tween, samples were incubated with the PLA-probes (Duolink In Situ PLA Probes anti-mouse plus, #DUO92001 and anti-rabbit minus, #DUO92005, Sigma-Aldrich) for 1 h at 37 °C, washed and ligated for 30 min at 37 °C. After additional washing steps the amplification with polymerase took place for 100 min at 37 °C. After staining the nuclei with DAPI the cells were washed several times and stored in PBS with 0.02% sodium azide. Images were acquired with a Zeiss LSM800 laser scanning microscope.

### siRNA transfection

Human aortic smooth muscle cells (HAoSMC) were seeded for 50% confluence and transfected the next day with Lipofectamine 3000 in 8% FCS (without antibiotics) using 40 nM siRNA for 72 h, with a medium change after 24 h. Scrambled Stealth RNAi™ (siScr1-3) was used as negative control (Supplemental Table 1). 4 h prior to experiments, the medium was changed to SMC basal medium from PELOBiotech (#PB-MH-200-2190) without supplements but with 1% FCS.

### Protein isolation and western blot analysis

Cells were washed twice with Hank’s buffer and lysed with Triton X-100 lysis buffer (20 mM TRIS/Cl pH 7.5, 150 mM NaCl, 10 mM NaPi, 20 mM NaF, 1% Triton X-100, 2 mM orthovanadate (OV), 10 nM okadaic acid, protein-inhibitor mix (PIM), 40 μg/ml phenylmethylsulfonyl fluoride). Cells were centrifuged for 10 min at full speed at 4° C. Supernatant was used and after determination of protein concentration by the Bradford assay, equal amounts of protein were boiled in sample buffer and separated by SDS-PAGE gel electrophoresis. The gels were blotted onto a nitrocellulose membrane and blocked in Rotiblock (Carl Roth, Germany). Infrared-fluorescent-dye-conjugated secondary antibodies were purchased from Licor (Bad Homburg, Germany) and detected with an infrared-based laser scanning detection system (Odyssey Classic, Licor, Bad Homburg, Germany). Used antibodies listed in Supplemental Table 4.

### Nuclear extraction

Cells were washed twice with Hank’s buffer and detached by scraping. After centrifugation, the pellet was resuspended in buffer A (10 mM Hepes pH 7.9, 10 mM KCl, 0.1 mM EDTA, 0.1 mM EGTA) and incubated for 15 min on ice. Subsequently, 0.75% Nonidet (NP-40) was added and the samples centrifuged at full speed for 1 min at 4 °C. The supernatant was removed as cytosolic fraction. The nuclear fraction was resuspended in buffer C (20 mM Hepes pH 7.9, 0.4 M NaCl, 1 mM EDTA, 1 mM EGTA) additionally with 6.25 units/mL Benzonase and incubated for 15 min at 25 °C while shaking. Afterwards the lysates were incubated for 1 h at 4 °C. After centrifugation 5 min full speed at 4 °C the nuclear fraction and the pellet (chromatin fraction) were isolated. Used antibodies listed in Supplemental Table 4.

### Electrophoretic mobility shift assay (EMSA)

Human aortic smooth muscle cells were stimulated with 100 nmol/L AEA, 150 min and subsequently treated with 10 ng/mL IL-1β, 30 min. Afterwards protein was isolated by nuclear extraction as described before. 5 µg nuclear protein was incubated for 30 min with IRDye pre-labeled oligonucleotides specific for NF-kB consensus sequence (#829-07924, LI-COR). Therefore supplements and instructions of the EMSA kit (#829-07910, LI-COR) were used. Samples were loaded on 5% native acrylamide gels and separated for 30 min.

### ELISA

Cells were treated as described before for protein isolation. The supernatant was collected after 4 h. Afterwards the ELISA was performed as written in the instructions of CCL2 ELISA Kit (#BMS281, Thermofisher).

### Quantitative RT-qPCR

Total RNA was extracted with an RNA Mini Kit (Bio&SELL). RNA from murine and human aortic tissue was extracted with a specific lysis buffer (4 M Guanidinium thiocyanate, 25 mM Na-citrat), 0.5% *N*-lauryl sarcosine and *β*-mercaptoethanol). The tissue was homogenized with a TissueLyser LT (Qiagen) three times for 1 min each and then centrifuged for 1 min at full speed. Subsequently the supernatant was further processed with the RNA Mini Kit (Bio&SELL). cDNA was prepared with SuperScript III reverse transcriptase (Thermo Fisher) and random hexamer (Promega, #C118A) together with oligo (dT) primers (Sigma-Aldrich, #O4387). Quantitative real-time PCR was performed with SYBR Green Master Mix and ROX as reference dye (Bio&SELL #76.580.5000) in AriaMX Cycler (Agilent). Relative expression of target genes was normalized to GAPDH (or *α*-actin for tissue samples) and analyzed by the delta-delta Ct method with the AriaMX qPCR software (Agilent Technologies, Santa Clara, CA, USA) (qRT-PCR primers: Supplemental Table 2).

### LC–MS/MS

Endocannabinoids and endocannabinoid-like substances were analyzed by liquid chromatography-electrospray ionization-tandem mass spectrometry (LC–ESI–MS/MS). Minor modifications were made due to the special requirements of tissue samples. In brief, tissue samples were homogenized in water/ethanol 3:1 (v/v) using a Qiagen tissue lyser, resulting in tissue homogenates of 0.04 mg/µL. The analytes were extracted by liquid–liquid-extraction with ethyl acetate/hexane 9:1 (v/v) using 200 μL tissue homogenate (8 mg tissue) after spiking with the respective isotopically labeled internal standards. Chromatographic separation of the analytes was done with an Agilent 1290 Infinity I UHPLC system equipped with an Acquity UPLC BEH C18 column (100 × 2.1 mm, 1.7 μm, Waters, Eschborn, Germany). The quantification of all analytes was carried out in a hybrid triple quadrupole-ion trap mass spectrometer QTRAP 6500+ (Sciex, Darmstadt, Germany) equipped with a Turbo-V-source operating in positive ESI mode. For all analytes, the concentrations of the calibration standards, quality controls and samples were evaluated by Analyst software 1.6.3 and MultiQuant software 3.0.2 (Sciex) using the internal standard method (isotope-dilution mass spectrometry). Calibration curves were calculated by linear regression with 1/*x *weighting.

### RNA sequencing

Total RNA was isolated with the RNA Mini Kit from Bio&SELL and on-column DNase digestion (DNase-Free DNase Set, Promega) was performed to remove any potential DNA contamination. Total RNA and library integrity were verified with LabChip Gx Touch 24 (Perkin Elmer). 1 µg of total RNA was used as input for SMARTer Stranded Total RNA Sample Prep Kit—HI Mammalian (Clontech) following standard instructions. Sequencing was performed on a NextSeq500 instrument (Illumina) with v2 chemistry, resulting in average of 38 M reads per library with 1 × 75 bp single end setup. The resulting raw reads were assessed for quality, adapter content and duplication rates with FastQC (Andrews S. 2010, FastQC: a quality control tool for high throughput sequence data. Available online at: https://www.bioinformatics.babraham.ac.uk/projects/fastqc). Trimmomatic version 0.33 was employed to trim reads after a quality drop below a mean of Q20 in a window of five nucleotides (Bolger et al., Trimmomatic: a flexible trimmer for Illumina sequence data). Only reads between 30 and 150 nucleotides were cleared for further analyses. Trimmed and filtered reads were aligned versus the Ensemble human genome version hg38 (GRCh38) using STAR 2.5.2b with the parameter “–outFilterMismatchNoverLmax 0.1” to increase the maximum ratio of mismatches to mapped length to 10% (Dobin et al., STAR: ultrafast universal RNA-seq aligner). The number of reads aligning to genes was counted with feature Counts 1.4.5-p1 tool from the Subread package [16]. Only reads mapping at least partially inside exons were admitted and aggregated per gene. Reads overlapping multiple genes or aligning to multiple regions were excluded. Differentially expressed genes were identified using DESeq2 version 1.62 [17]. Adjusted *p* values were determined by Benjamini–Hochberg correction with a *p* value of 0.05 considered significant. The Ensembl annotation was enriched with UniProt data (release 06.06.2014) based on Ensemble gene identifiers (Activities at the Universal Protein Resource (UniProt)). The heatmap shows the *Z* score of each individual replicate of each condition. The *Z* score was calculated across all replicates for each gene from log-normalized expression. All in the heatmap represented genes are listed in the supplemental Table 5.

### ATAC sequencing

Cells were trypsinized and washed with PBS. Washed cells were counted and 50.000 cells were used for ATAC Library preparation using Tn5 Transposase from Nextera DNA Sample Preparation Kit (Illumina). Cell pellet was resuspended in 50 µl PBS and mixed with 25 µl TD-Buffer, 2.5 µl Tn5, 0.5 µl 10% NP-40 and 22 µl water. Cell/Tn5 mixture was incubated at 37 °C for 30 min with occasional snap mixing. Transposase treatment was followed by 30 min incubation at 50 °C together with 500 mM EDTA pH8.0 for optimal recovery of digested DNA fragments. For neutralization of EDTA 100 µl of 50 mM MgCl2 was added followed by purification of the DNA fragments by MinElute PCR Purification Kit (Qiagen). Amplification of Library together with Indexing was performed as described elsewhere [3]. Sequencing, mapping, and read filtering: libraries were mixed in equimolar ratios and sequenced on NextSeq500 platform using V2 chemistry with paired-end mode following assessment for quality using FastQC (Andrews S. 2010, FastQC: a quality control tool for high throughput sequence data. Available online at: https://www.bioinformatics.babraham.ac.uk/projects/fastqc). Trimmomatic version 0.33 was employed to trim reads after a quality drop below a mean of Q20 in a window of five nucleotides [2]. Only reads above 30 nucleotides were cleared for further analyses. Reads were mapped versus the hg19 version of the human genome with STAR 2.4.2a [7] using only unique alignments to exclude reads with unclear placing. The reads were further deduplicated using Picard 1.136 (Picard: A set of tools (in Java) for working with next generation sequencing data in the BAM format; https://broadinstitute.github.io/picard/) to avoid PCR artifacts leading to multiple copies of the same original fragment. Peak calling, filtering, and annotation: For identification of peaks the MUSIC peakcaller (version from December 2015) [9] was employed in punctate mode to accommodate for the range of peak widths typically expected for ATAC-seq. Unification of peaks: to compare peaks in different samples, the resulting lists of significant peaks were overlapped and unified to represent identical regions. After conversion of BAM files to BigWig format with deepTools bamCoverage [28], the counts per unified peak per sample were computed with BigWigAverageOverBed (UCSC Genome Browser Utilities, https://hgdownload.cse.ucsc.edu/downloads.html). Raw counts for unified peaks were submitted to DESeq2 for normalization [1]. Spearman correlations were produced to identify the degree of reproducibility between samples using R. Normalization of samples for IGV: to permit a normalized display of samples in IGV, the raw BAM files were normalized for sequencing depth (number of mapped deduplicated reads per sample) and noise level (number of reads inside peaks versus number of reads not inside peaks). Two factors were computed and applied to the original BAM files using bedtools genomecov resulting in normalized BigWig files for IGV.

### Statistics

Unless otherwise indicated, data are given as means ± standard error of mean (SEM). Calculations were performed with Prism 8.0 or BiAS.10.12. For multiple group comparisons, ANOVA followed by Tukey’s or Sidak’s multiple comparison was performed. Data without normal distribution were tested with nonparametric ANOVA followed by Kruskal–Wallis test and Dunn’s correction. Individual statistics of unpaired samples were performed by unpaired *t* test, if not normally distributed with Mann–Whitney test. *p* values of < 0.05 were considered as significant. Unless otherwise indicated, *N* indicates the number of individual experiments.

### Source of founding

This work was supported by grants from the DFG, SFB1039 (TP A01 (RPB), A02 (DS), A06 (IF), B07 (DMzH) and Z01 (GG)), by the Cardio-Pulmonary Institute—CPI and by the Goethe University Frankfurt, Germany.

### Data availability

We have uploaded the RNA-Seq data into the public NCBI GEO database (https://www.ncbi.nlm.nih.gov/geo/query/acc.cgi?acc=GSE131732).

## Results

### AEA attenuates a subset of IL-1β responsive genes in HAoSMC

**Fig. 1:**
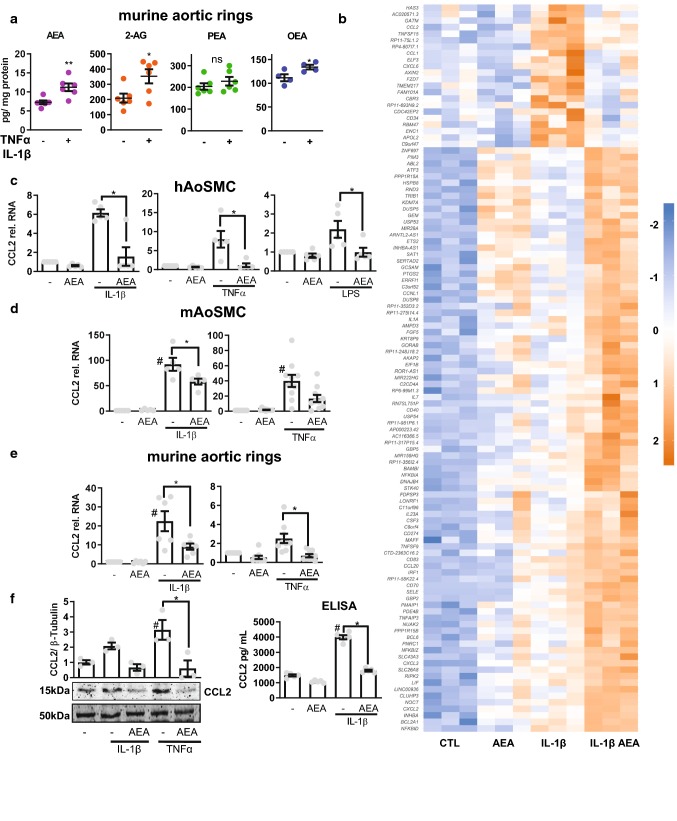
Anandamide (AEA) is released by vascular cells and attenuates induction of a subset of IL-1β responsive genes in HAoSMC. **a** Cellular concentration of the indicated endocannabinoids as determined by LC–MS/MS from murine aortic rings treated with TNFα and IL-1β 10 ng/mL each, 4 h or with control (PBS with 0.1% BSA). *N* = 4–6, unpaired *t* test. **b** Heatmap showing *Z* score of restricted to IL-1β-induced genes with expression significantly altered by 100 nmol/L, 150 min AEA and subsequently 10 ng/mL IL-1β for 90 min in HAoSMC. *N* = 3 RT-qPCR of CCL2 performed from human aortic (**c**) and mouse aortic (**d**) smooth muscle cells and murine aortic rings (**e**) in response to the stimuli indicated. Cells treated with 100 nmol/L AEA for 150 min (for tissue: overnight) and subsequently stimulation with 10 ng/mL cytokine for 90 min. *N* = 5–10 **f** Protein levels of CCL2 as determined by Western blot analysis after treatment with 100 nmol/L AEA for at least 1 h and subsequently 10 ng/mL IL-1β for 3 h (normalized to *β*-tubulin, *N* = 3) or ELISA *N* = 5. Ordinary one-way ANOVA with Tukey’s multiple comparison post-hoc test. If not normally distributed nonparametric ANOVA with Kruskal–Wallis test and Dunn’s multiple comparison post-hoc test

To infer an autocrine or paracrine function, the vascular production of endocannabinoids should be dynamic. To test this, murine aortic rings were stimulated with TNFα and IL-1β or solvent for 4 h and endocannabinoid production was measured by LC–MS/MS **(**Fig. 1a). Stimulation with the combination of TNFα and IL-1β cytokines increased the production of AEA and 2-AG as well as OEA, but not PEA in murine aortic tissue, with AEA levels increasing the most significant (Fig. 1a).

Therefore, all subsequent studies focused on this highly important endocannabinoid. To determine whether AEA is relevant for cellular gene expression, human aortic smooth muscle cells (HAoSMC) were pre-stimulated with the endocannabinoid for 150 min followed by cytokine stimulation for 90 min and subsequent gene expression analysis by RNA-Seq. This protocol was chosen because 90 min cytokine stimulation was unable to significantly increase endocannabinoid production (data not shown). Interestingly, AEA had a profound effect on cytokine-mediated gene induction: IL1β induced 409 genes in HAoSMC and AEA potentiated the induction of 92 of them. The induction of 21 IL1β-responsive genes was suppressed by AEA (Fig. 1b, top regulated genes; Table 1), among these genes were several important pro-inflammatory cytokines like CXCL6, CCL1 and CCL2 [33]. This suggests that AEA impacts inflammatory signaling and chemokine induction in particular, which is reflected by the gene ontology (GO) analysis (Tables 2, 3).

**Table 1:** List of AEA affected genes

ENSEMBLE	Gene	Log2 fold change	*p* value
ENSG00000108702	CCL1	− 2.979705102	0.011806624
ENSG00000235505	CASP17P	− 2.846548032	0.005539894
ENSG00000163435	ELF3	− 2.292228757	0.037224264
ENSG00000103044	HAS3	− 1.640698004	0.004274255
ENSG00000174059	CD34	− 1.604414104	0.0275882
ENSG00000168646	AXIN2	− 1.567410529	0.012764475
ENSG00000172738	TMEM217	− 1.376487678	0.002107083
ENSG00000155760	FZD7	− 1.28979261	1.18E−06
ENSG00000163694	RBM47	− 1.271884075	0.046690067
ENSG00000171766	GATM	− 1.270319882	0.011426252
ENSG00000124875	CXCL6	− 1.263261284	0.044658625
ENSG00000181634	TNFSF15	− 1.262629814	0.006991752
ENSG00000255521	AL356215.1	− 1.257583402	0.010741181
ENSG00000108691	CCL2	− 1.247502441	0.010281235
ENSG00000229056	HECW2-AS1	− 1.120083132	0.041257119
ENSG00000178882	RFLNA	− 1.038896509	0.046389575
ENSG00000213443	AC007068.1	− 0.952963163	0.026106235
ENSG00000149798	CDC42EP2	− 0.923597937	0.045922029
ENSG00000128335	APOL2	− 0.865889174	0.00824209
ENSG00000171617	ENC1	− 0.86382712	0.002088145
ENSG00000159231	CBR3	− 0.756175822	0.039504656

**Table 2:** Go ontology analysis of AEA affected genes in context to linked diseases

Index	Disease		*p* value
1	Inflammation		0.00006722
2	Diffuse cutaneous leishmaniasis		0.0001415
3	Lung injury		0.0001972
4	Leukemia, mast-cell		0.0002621
5	Chronic lung injury		0.0003361
6	Angiofibroma		0.0003904
7	Thyroid diseases		0.0003986
8	Bright disease		0.0004487
9	Pulmonary hypertension		0.000498
10	Nephritis, interstitial		0.000511

**Table 3:** Go ontology analysis of AEA affected genes on their biological processes

Index	Biological process	*p* value
1	Chemokine-mediated signaling pathway (GO:0070098)	0.00001674
2	Neutrophil chemotaxis (GO:0030593)	0.00002011
3	Mmulticellular organism development (GO:0007275)	0.00004792
4	Chemotaxis (GO:0006935)	0.00006417
5	Monocyte chemotaxis (GO:0002548)	0.0007215
6	Cellular response to interferon-gamma (GO:0071346)	0.001012
7	Inflammatory response (GO:0006954)	0.001302
8	Cellular response to interleukin-1 (GO:0071347)	0.001401
9	Cellular response to lipopolysaccharide (GO:0071222)	0.001508
10	Signal transduction (GO:0007165)	0.001672

We chose to investigate this further given the importance of CCL2 release from VSMC in aneurysm and arteriosclerosis development [13]. AEA not only blocked the IL1β-mediated CCL2 induction but also TNFα- and LPS-mediated induction, as determined by qRT-PCR (Fig. 1c). Moreover, CCL2 induction was suppressed in both the murine aortic smooth muscle cells (Fig. 1d) and in the intact murine aorta tested by ex vivo experiments (Fig. 1e). Furthermore, AEA prevented cytokine-mediated CCL2 protein induction and secretion, as determined by Western blot and ELISA respectively (Fig. 1f).

### AEA blocks the IL-1β-induced leukocyte attraction

**Fig. 2:**
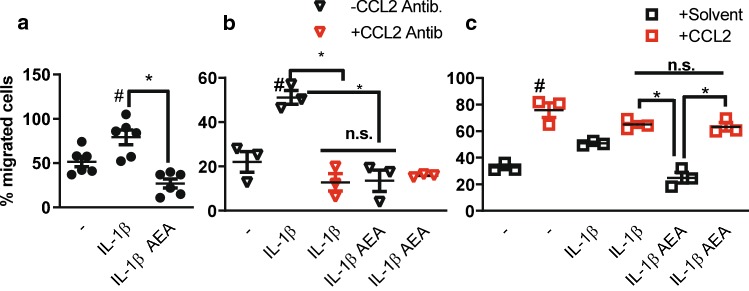
CCL2 is a functional important target of the inhibitory effect of AEA. **a**–**c** Boyden chamber assay with THP1 leukocytic cells and conditioned medium of HAoSMCs treated with or without 100 nmol/L AEA (2 h) and 10 ng/mL IL-1β (3 h) as indicated (**a**). Effect of CCL2-neutralizing antibody (**b**, 1:1000 in condition medium**)** or CCL2 protein recombinant protein (**c**, 50 ng/mL in conditioned medium). *N* = 3–6, ^#^*p* < 0.05—vs. treatment. Ordinary one-way ANOVA with Tukey’s multiple comparison post-hoc test

Since AEA only affected a subset of cytokine-induced genes, we wondered whether the effect was functionally important. Therefore, a Boyden chamber assay was performed to study leukocyte-migration towards smooth muscle cell-conditioned medium. Medium from IL-1β-conditioned SMC elicited a stronger leukocyte attraction than control medium, and this effect was lost after pre-stimulation of the SMC with AEA (Fig. 2a). Importantly, a CCL2-blocking antibody added to the conditioned medium obtained from SMC stimulated with IL-1β prevented the pro-migratory effect but did not alter that of conditioned medium obtained from SMC pretreated with AEA prior to IL-1β stimulation (Fig. 2b). Finally, addition of recombinant CCL2 to conditioned medium obtained from SMC pretreated with AEA prior to IL-1β stimulation elicited a similar pro-migratory effect as with conditioned medium from IL-1β stimulated SMC. Collectively, these data demonstrate that the effect of AEA is highly important biologically and is mediated by CCL2.

### AEA-mediated anti-inflammatory actions are not caused by altered NF-κB/AP-1 signaling

**Fig. 3:**
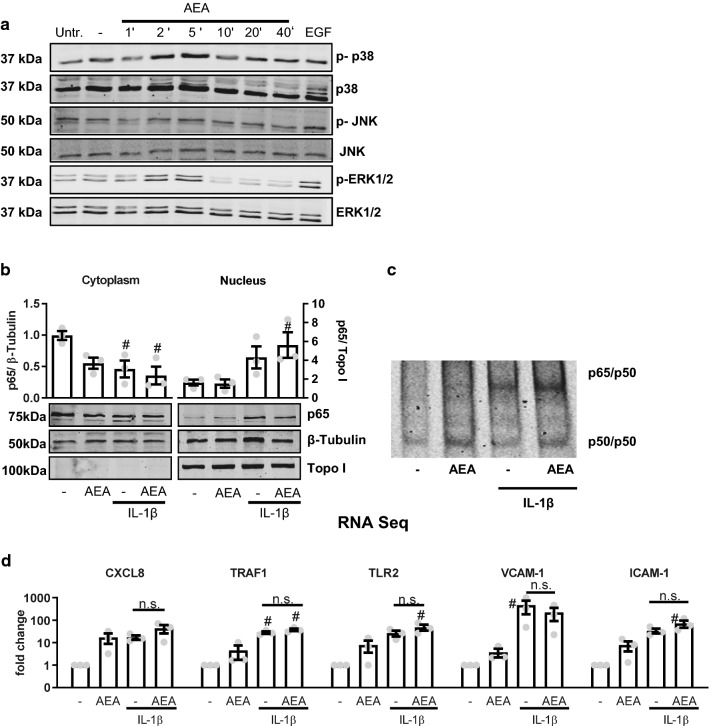
AEA-mediated anti-inflammatory actions are not mediated by altered NF-kB/AP-1 signaling. **a** Effect of AEA (100 nmol/L, applied for the minutes indicated) and the positive control EGF (10 ng/ml, 10 min) on expression and phosphorylation (indicated by *p*) of the MAP kinases shown as detected by Western blot from HAoSMC. *Untr.* untreated, *-* solvent control (ethanol 0.05%). **b** Western blot analysis of NF-κB p65 localization in HAoSMC after exposure to IL1β (30 min, 10 ng/ml) and AEA (150 min, 100 nmol/L). *N* = 3, **c** Exemplary electrophoretic mobility shift assay (EMSA) for NFκB of human aortic smooth muscle cells treated with or without AEA (CTL or 100 nmol/L, 150 min) and subsequently with our without IL-1β (10 ng/mL, 30 min). **d** Expression of known NF-κB/AP-1 target genes from the RNA-Seq data set. Reads are normalized to solvent control to determine fold changes shown on a logarithmic axis. *N* = 3. ^#^*p* < 0.05—vs. treatment. Ordinary one-way ANOVA with Tukey’s multiple comparison post-hoc test. If not normally distributed nonparametric ANOVA with Kruskal–Wallis test and Dunn’s multiple comparison post-hoc test

It is well known that cytokines like TNFα and IL-1β induce inflammatory gene expression through activation of NF-κB or AP-1. Moreover, it has been shown that NF-κB and AP-1 are both crucial for CCL2 gene expression [35]. Gene ontology analysis of the 21 AEA-sensitive and IL-1β-induced genes recovered a signature containing the RELA subunit of NF-κB as well as FOS of the AP-1 transcription factors (data not shown). We therefore investigated whether AEA impacts NF-κB or AP-1 signaling. Compatible with a cannabinoid receptor-dependent activation of G_i_-proteins, AEA acutely increased the phosphorylation of p38-MAP Kinase and ERK1/2 (Fig. 3a) but had no effect on the cytokine-induced translocation of the NF-κB subunit p65 (RELA) into the nucleus (Fig. 3b) or on the formation of the NFκB p50/p65 complex in the nucleus as determined by EMSA (Fig. 3c). These data suggest that AEA increases rather than attenuates the induction of NF-κB and AP-1-dependent genes. To address this, the RNA-Seq data were screened for genes which are known to be regulated by these transcription factors: CXCL8 [14], TRAF1 [29], TLR2 [13], VCAM-1 [12] and ICAM-1 [4]. As shown in Fig. 3d, most of these genes were induced by AEA itself and AEA did not block the IL-1β-mediated induction. Collectively, these data suggest that direct inhibition of AP-1 or NF-κB is unlikely to be the mechanism through which AEA mediates its anti-inflammatory effect.

### AEA treatment inhibits CCL2 expression through HDAC4-dependent gene repression

**Fig. 4:**
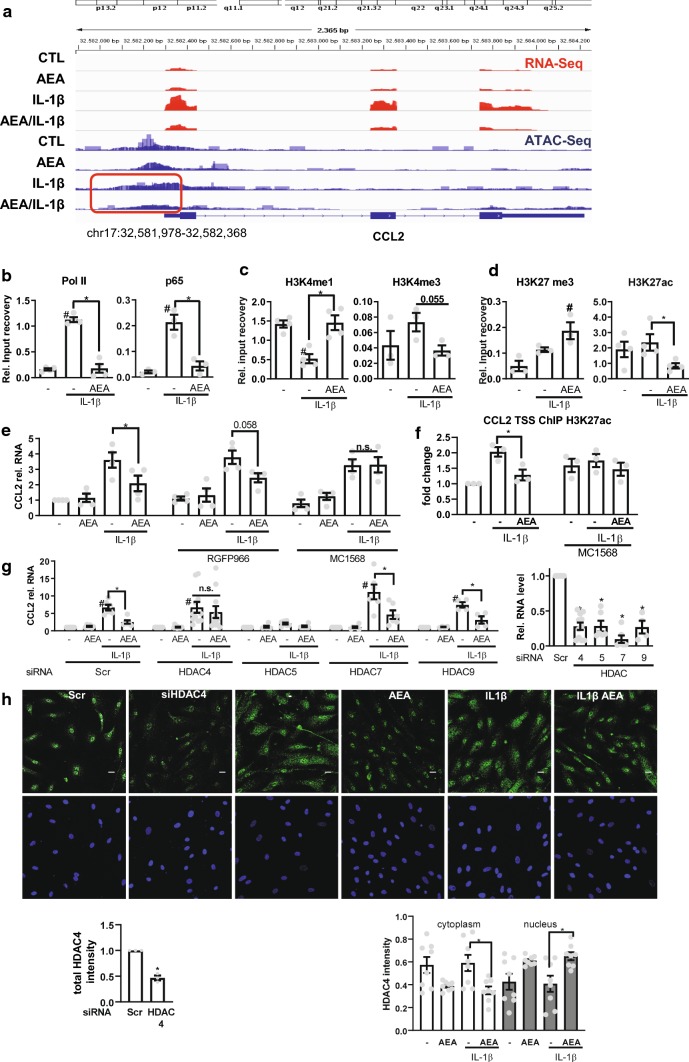
AEA-induced CCL2 repression involves HDAC4. **a** RNA-Seq and ATAC-Seq channel overly tracing of HAoSMC for the stimuli indicated (100 nmol/L AEA, 150 min und 10 ng/mL, IL-1β 90 min). Focusing chromatin structure at the CCL2 Promoter region chr17:32,581,978-32,582,368 in context to IL-1β ± AEA treatment (red box). *N* = 3 Chromatin immunoprecipitation (ChIP) of the proteins indicated followed by qPCR for the CCL2 transcription start site (TSS) in the absence (**b**–**d**) or presence (**f**) of MC1568 (32 µM). **e** CCL2 RT-qPCR of HAoSMC pretreated with the HDAC inhibitor RGFP966 (2.7 µM) or MC1568 (32 µM) or solvent (DMSO) for 1 h and afterwards stimulated with AEA (100 nmol/L 150 min) and IL-1β (10 ng/mL 90 min; for ChIP experiments: 120 min 100 nmol/L AEA and 10 ng/mL IL-1β 60 min). Two-way ANOVA with Tukey’s multiple comparison post-hoc test. **g** CCL2 (left), HDAC (right) RT-qPCR after siRNA knockdown of the HDACs indicated (40 nM, 72 h). Normalized to each siRNA untreated (−). *N* = 4–11, two-way ANOVA with Tukey’s multiple comparison post-hoc test. **h** Immunofluorescence of HDAC4 in HAoSMC after treatment with AEA (100 nmol/L, 150 min) and IL-1β (10 ng/mL, 60 min) or HDAC4 siRNA. Scale bar 20 µm, *N* = 8. Immunofluorescence after siRNA knockdown (40 nM, 72 h) *n* = 3, unpaired *t* test. ^#^*p* < 0.05—vs. treatment, *p < 0.05. Ordinary one-way ANOVA with Tukey’s multiple comparison post-hoc test. If not normally distributed nonparametric ANOVA with Kruskal–Wallis test and Dunn’s multiple comparison post-hoc test

Since AEA did not interfere with AP-1 or NF-κB signaling, it might suppress binding of NF-κB to DNA epigenetically. To investigate the accessibility of chromatin at the CCL2 promoter, an assay for Transposase-accessible chromatin using sequencing (ATAC-Seq) was performed. Indeed ATAC-Seq demonstrated that IL-1β induced accessible DNA regions at the CCL2 promoter and that AEA in contrast compacts the chromatin (Fig. 4a).

This was studied further with chromatin immuno-precipitation (ChIP) of the CCL2 promoter. As expected, IL-1β stimulation increased binding of RNA polymerase II and NF-kB p65 to the CCL2 transcriptional start site, in turn activating CCL2 transcription (Fig. 4b). Moreover, histone 3 lysine 4 monomethylation (H3K4me1), which marks promoter inactivation [5] was decreased and H3K4 trimethylation (H3K4me3), a mark for transcriptional activation [32] was increased (Fig. 4c). Importantly, all of these IL-1β mediated effects were prevented by AEA pretreatment. Given that nuclear import of NF-κB was unaffected and that AP-1 target genes were increased with AEA, it would appear that AEA instead activates an independent epigenetic mechanism. We, therefore, investigated the CCL2 H3K27 modification landscape. Interestingly, while IL1β had little impact on the modification of H3K27 at the CCL2 TSS, AEA pretreatment increased H3K27me3, a mark for heterochromatin [31] and simultaneously decreased H3K27 acetylation, a mark for promoter activation (Fig. 4d). This suggests that AEA treatment potentially results in the recruitment of a histone deacetylase (HDAC) to the CCL2 promoter, which leads to epigenetic gen**e** silencing.

To determine whether the observed effect was indeed mediated by HDACs, we first used HDAC inhibitors and measured CCL2 expression. RGFP966, an HDAC class I inhibitor [20], had no effect on AEA-induced CCL2-repression, while MC1568, an HDAC class IIa inhibitor [8], completely blocked the AEA-mediated repression of CCL2 (Fig. 4e) as well as the AEA-mediated deacetylation of H3K27 (Fig. 4f). The HDAC IIa family consists of four members (HDAC 4, 5, 7 and 9). To determine the specific HDAC responsible for this effect, knockdown experiments with siRNAs against each HDAC class IIa member were performed. Treatment with each siRNA resulted in a more than 70% reduction in the respective HDAC mRNA expression (Fig. 4g). However, only knockdown of HDAC4 blocked the effect of AEA on CCL2 mRNA expression, whereas knockdown of HDAC5 completely blocked the inflammatory response. AEA treatment resulted in the nuclear accumulation of HDAC4 (Fig. 4h) which is in agreement with the fact that class IIa HDACs shuttle between the cytoplasm and nucleus depending on their activation [11]. Together, these data suggest that AEA facilitates nuclear import of HDAC4 and its recruitment to the CCL2 gene locus, where it mediates gene repression.

### AEA prevents removal of NCoR1 from the CCL2 promoter

**Fig. 5:**
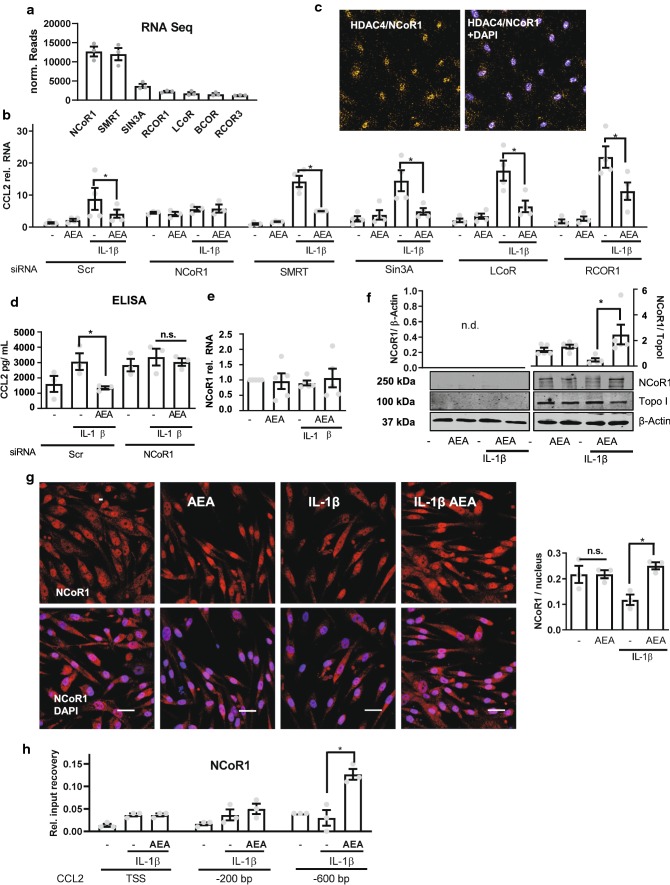
NCOR1 mediates AEA-induced CCL2 suppression. **a** mRNA expression of repressors in HAoSMC from the RNA-Seq dataset normalized by median of ratios. *N* = 3. **b** CCL2 RT-qPCR in HAoSMC after siRNA knockdown of the repressors indicated (40 nM, 72 h). Scr denotes the scrambled siRNA control. Normalized to siRNA Scr. Treatment: 100 nmol/L AEA, 150 min and IL-1β 10 ng/mL 90 min. *N* = 3–4, two-way ANOVA with Tukey’s multiple comparison post-hoc test. **c** Proximity ligation assay in HAoSMC for HDAC4 and NCoR1 after treatment 100 nmol/L AEA, 150 min and 10 ng/mL IL-1β 60 min, *N* = 4. **d** CCL2 protein levels measured by ELISA with and without siRNA-mediated knockdown of NCoR1 or Scr as control. Media was collected after 4 h as described before. Two-way ANOVA with Tukey’s multiple comparison post-hoc test. **e** NCoR1 expression as determined by RT-qPCR of HAoSMC. *N* = 5. **f** Western blot analysis of HAoSMC nuclear extracts for NCoR1 after treatment with 100 nmol/L AEA 150 min and 60 min 10 ng/mL IL-1β. *N* = 5. **g** Immunofluorescence of NCoR1 in HAoSMC treated similar to (**f**). Scale bar = 50 µm, *N* = 4. **h** ChIP analysis of NCoR1 in HAoSMC *n* = 4. Treatment 120 min 100 nmol/L AEA and 10 ng/mL IL-1β 60 min. *p < 0.05. Ordinary one-way ANOVA with Tukey’s multiple comparison post-hoc test. If not normally distributed nonparametric ANOVA with Kruskal–Wallis test and Dunn’s multiple comparison post-hoc test

HDACs are normally recruited to genes by repressors or corepressor complexes [21]. To identify the repressive factor required for AEA-mediated anti-inflammatory actions and HDAC4 recruitment, the RNA-Seq data was reanalyzed for the expression of proteins participating in repressive epigenetic events in HAoSMCs. Several repressors were detected, with NCoR1 and SMRT exhibiting the highest mRNA abundance (Fig. 5a). Knockdown of the five most abundant repressors by siRNA revealed that NCoR1 is the repressor involved in CCL2 expression (Fig. 5b). Its knockdown increased basal CCL2 expression, completely blocked the inflammatory response and prevented the inhibitory effect of AEA. In contrast, knockdown of the other repressors slightly increased the IL-1β response but had no effect on basal CCL2 expression or the inhibitory effect of AEA. Proximity ligation assays indeed demonstrated a co-localization of NCoR1 and HDAC4 (Fig. 5c). Furthermore, ELISA measurements revealed that NCoR1 knockdown increased basal CCL2 secretion, prevented the effect of IL-1β on CCL2 and blocked the anti-inflammatory effect of AEA (Fig. 5d).

These observations suggest that under basal conditions the CCL2 promoter is suppressed by NCoR1 and that IL-1β reduces this binding which can be prevented by AEA. To test this, the expression and localization of NCoR1 was studied. Neither AEA nor IL-1β influenced NCoR1 mRNA expression (Fig. 5e). However, IL-1β promoted NCoR1 nuclear export and this effect was prevented by AEA, as observed by Western blot analysis and immuno-fluorescence (Fig. 5f, g). Finally, we wanted to perform NCoR1 immuno-precipitation followed by protein interaction analyses and mass spectrometry. Unfortunately, pulldown of NCoR1 with all available antibodies yielded unsatisfactory results for co-immunoprecipitation experiments. Nevertheless, NCoR1 ChIP revealed the putative site of action on the CCL2 promoter demonstrating that NCoR1 binds to a region 600 bp upstream of the CCL2 transcriptional start site. This interaction was potentiated by AEA treatment (Fig. 5h).

## Discussion

In the present work we demonstrate a novel suppressive effect of the endocannabinoid AEA on a specific subset of inflammatory genes. Pretreatment with AEA elicited a strong anti-inflammatory response through recruitment of the NCoR1 corepressor complex to the CCL2 locus. Furthermore, AEA promoted the nuclear import of HDAC4 which resides in the nucleus near NCoR1. Thus, in response to AEA, the nuclear corepressor NCoR1 is retained and stabilized at the promoter of CCL2, resulting in suppression of cytokine-induced gene expression by an HDAC4-dependent deacetylation of H3K27.

Despite being somewhat indirect, the interaction between a repressor and HDACs seems to be a fundamental mechanism of gene expression. Although we could demonstrate a close proximity between HDAC4 and NCoR1 in the nucleus, we failed to demonstrate a direct protein–protein interaction of the two proteins by co-immuno-precipitation or immuno-precipitation followed by mass spectrometry (data not shown). Interestingly, it has recently been suggested in cardiac myocytes that interaction of the two proteins is indeed mediated by a third protein, a member of the MEF2 family [15]. In our own mass spectrometry analysis, we were unable to recover MEF2 as an NCoR1 interaction partner. It therefore seems plausible that a so far unidentified transcription factor mediates this interaction in SMCs. This aspect is likely to be important as AEA only suppressed a subset of IL-1β inducible genes. The ATAC-Seq data suggested that AEA selectively induced chromatin compaction on these genes (like CCL1, CXCL6 and CCL2), whereas chromatin was observed to be open for the genes induced by AEA, like CXCL8 and VCAM-1. It is tempting to speculate that this difference is mediated by binding of NCoR1 to the DNA. This, however, is difficult to demonstrate, as NCoR1 does not directly bind DNA and thus does not contain a defined DNA-binding motif. Additionally, numerous transcription factors can participate in DNA binding of a nuclear repressor complex. Despite these considerations, NCoR1 ChIP-Seq data has been published (Encode: GSM1003565). Although the quality of this data is suboptimal, due to the points mentioned above, decoration of CCL2, CXCL6 and CCL1 by NCoR1 was observed. Other genes which were unaffected by AEA pretreatment did not contain a clear NCoR1 signature in their promoter region.

In addition to the anti-inflammatory effect acting on CCL2 and some other genes, AEA increased the expression of some other inflammatory genes (see heat map). Some of them contain AP-1 and NFκB binding sites and thus it appears that the function of AEA depends on the gene context. The pro-inflammatory function of AEA is not mediated by NCoR-1 (data not shown) so that we did not address further this aspect in the present work.

In the present study, we could demonstrate that AEA is released from the murine aorta in response to cytokine stimulation and that exogenously-applied AEA strongly prevents cytokine-mediated CCL2 induction. This mechanism strongly suppresses cytokine-mediated CCL2 induction and thereby prevents leukocyte chemotaxis. It is, however, unclear whether the endogenous production of AEA is sufficient to elicit a similar response. Although AEA is considered a very important endocannabinoid, it is somewhat unstable due to its metabolism by several enzymes. It is believed that AEA is formed from *N*-arachidonoyl-phosphatidylethanolamine (NAPE) by the action of NAPE-PLD. Besides this there is evidence that AEA is also formed by enzymatic activity of other enzymes like PLA_2_, PLC/PTPN22 and ABHD4/GDE1. NAPE itself is produced by several *N*-acyltransferases [19]. The redundancy in this pathway makes approaches with knockout mice specific for the individual enzyme unfeasible. The same limitation applies for AEA degradation, which can occur through many different pathways involving the P450 monooxygenases and lipoxygenases [19], which are all expressed in the vascular system. We were unable to increase vascular AEA levels using cyclooxygenase inhibition and knockdown cells targeting the degradation pathways to arachidonic acid by FAAH and NAAA (data not shown). Therefore, the present work reports a novel interesting pharmacological pathway but its exploitation will depend on the possibility to generate AEA mimetics.

In conclusion, we have identified a novel potent anti-inflammatory epigenetic mechanism for the endogenous endocannabinoid anandamide (AEA). AEA mimetics could be developed as potent tools to limit chemotactic recruitment of monocytic cells into areas of inflammation.

## Electronic supplementary material

Below is the link to the electronic supplementary material.

Supplementary file1 (PDF 514 kb)

Supplementary file2 (PDF 311 kb)
