# Supplementary material for: The endocannabinoid anandamide has an anti-inflammatory effect on CCL2 expression in vascular smooth muscle cells
Source: Basic Res Cardiol. 2020 Apr 22;115(3):34. doi: 10.1007/s00395-020-0793-3 (PMC7176595; doi:10.1007/s00395-020-0793-3)
Supplement: Supplementary file 1 — Supplementary file1 (PDF 514 kb) [file 395_2020_793_MOESM1_ESM.pdf]

Supplemental Table 5 **z-score of regulated genes shown in the heatmap**

Gene	ETOH_1	ETOH_2	ETOH_3	AEA_1	AEA_2	AEA_3	ETOH.il1b_1	ETOH.il1b_2	ETOH.il1b_3	AEA.il1b_1	AEA.il1b_2	AEA.il1b_3
NFKBID	-0.93226995	-1.256711756	-1.265158542	-0.603763988	-1.063434485	-0.166082359	0.886220946	0.500735739	0.322651032	1.298855603	1.183281335	1.095676426
BCL2A1	-0.95176363	-0.814587602	-2.065369012	-0.202913034	-0.452724999	-0.351554333	0.492224453	0.663111156	0.204612777	1.306787851	1.039277342	1.132899032
INHBA	-1.88244944	-0.872961997	-1.573129086	0.008189493	0.078159144	0.296106512	0.048322642	0.409267123	0.062045797	1.274544032	1.154168236	0.997737544
CXCL2	-1.519980804	-1.52784013	-1.239295447	0.157530878	0.03282959	0.679354562	-0.436054219	0.352736525	0.061574016	1.242164214	1.267903105	0.92907771
NOCT	-1.628042039	-1.179631157	-1.065670187	-0.718219006	-0.571952197	0.326848042	0.170937829	0.626360723	0.502852232	1.276149974	1.216512604	1.043853183
CLUHP3	-1.772890088	-1.243492994	-0.93901898	-0.343876509	-0.127096415	0.846143707	-0.342869914	-0.114581157	0.488918858	1.246907913	1.199334262	1.102521317
LINC00936	-1.291413172	-1.293899968	-1.032568235	-0.315385396	-0.486001565	0.827162599	-0.265798627	-0.419836908	0.353569484	1.118160565	1.322512024	1.483499199
LIF	-1.851372168	-1.074793209	-1.533485209	-0.225127899	0.039162556	0.561516111	0.044109168	0.785920171	0.30041869	0.815060511	1.205621829	0.932969449
RIPK2	-1.431039517	-0.96018118	-1.904473623	-0.172939221	0.088848568	-0.124827568	0.217510462	0.483449922	0.42513696	0.892900989	1.27097646	1.214637748
SLC26A8	-1.243014485	-0.894107391	-1.163589574	-0.956263987	-0.825522138	0.202971445	0.425353318	0.059254018	0.599045968	0.825346598	1.367113525	1.603412702
CXCL3	-1.595084753	-1.52983549	-1.486411307	-0.00054693	0.024883455	0.60519468	0.004402296	0.482468339	0.439811718	1.04827847	0.993679779	1.013159743
SLC43A3	-1.004568695	-1.007674936	-1.314457153	-0.206179415	-0.702633841	0.868382435	-0.516547705	-0.289887816	0.382570287	0.913912166	0.945549955	1.931534718
NFKBIZ	-1.721086117	-1.21506925	-1.086578652	-0.655686727	-0.445278224	0.533966094	0.06773342	0.591684571	0.56384137	1.018548878	1.150658279	1.197266357
PNRC1	-0.95668649	-1.286170532	-0.506091722	-0.493117717	-1.116627579	0.977192166	0.047054157	-0.710898431	0.394597437	1.483165373	0.575276855	1.592306483
BCL6	-1.173145606	-1.712990575	-1.188252158	0.353806707	-0.542002289	0.807672702	-0.052941211	-0.125851045	0.217766861	1.354935004	0.730174082	1.330827527
PPP1R15B	-1.319873996	-1.094035532	-1.345174128	-0.03416801	-0.316098571	0.35766464	-0.165120509	-0.393111132	0.270479191	1.713982193	0.91178158	1.413674275
NUAK2	-1.520308832	-1.592347068	-0.927411926	0.400750874	-0.523208024	0.840284119	-0.354320089	-0.113325497	0.46228264	1.378801558	0.959002692	0.989799554
TNFAIP3	-1.448462878	-1.324443518	-1.16295733	-0.374293077	-0.673841325	0.618301599	-0.10540813	0.365493102	0.468320514	1.377487922	1.084999622	1.174803499
PDE4B	-0.871329107	-1.350871279	-1.735969767	0.02878379	-0.933507303	0.149415728	0.27545455	0.436856815	0.639088587	1.327983836	1.02904726	1.00504689
PMAIP1	-1.386241777	-1.842695955	-1.024227259	0.500367747	-0.114998173	0.322133892	-0.293691308	-0.025312929	0.520668922	1.408987124	0.807764918	1.127244798
GBP2	-0.968196178	-1.150420578	-1.017109596	-1.04592969	-0.95048083	0.049370086	0.196098229	0.298218572	0.820117177	0.941972716	1.521224705	1.305135387
SELE	-1.064467124	-1.064648386	-1.064233639	-0.975119336	-1.064220909	0.024140074	0.128828001	0.650091802	0.779522843	1.112368465	1.34805444	1.18968377
CD70	-1.023966528	-1.024658758	-1.023075937	-0.87470768	-0.873611768	-0.252899347	0.135239468	0.133678218	0.638367121	1.379462326	1.524702038	1.261470848
RP11-58K22.4	-1.143333762	-0.992075126	-0.625824183	-0.736144045	-1.142330121	0.872167876	-0.132937165	-0.529007203	0.764187827	0.84680242	1.14572735	1.672766133
IRF1	-1.119083066	-1.369171862	-1.109431808	-0.933298653	-0.936218005	0.308315262	0.44819558	0.693382081	0.815690437	0.926275967	1.192363804	1.082980264
CCL20	-1.31881675	-0.916654245	-1.153248297	-1.096903774	-0.866015076	0.390836301	0.238310967	0.486397487	0.729517946	1.163050381	0.998210497	1.345314564
CD83	-1.138074305	-1.101630898	-1.256664641	-0.856446464	-1.012573552	-0.101963933	0.786742545	0.67489624	0.819387593	1.146666225	1.051379932	0.988281257
CTD-2363C16.2	-0.768124897	-1.605963805	-0.504392851	0.012898341	-1.28567804	0.489127643	-0.070054823	-0.633003633	0.688580924	1.126888408	0.898094498	1.651628237
TNFSF9	-1.118684011	-1.097233049	-1.295158304	-0.623597995	-0.909132873	-0.287715437	0.409641372	0.367719317	0.936009516	1.310654509	1.083785009	1.223711944
MAFF	-2.201929836	-0.531482508	-1.526635948	0.15537164	0.086969108	0.67903254	-0.095853941	0.127280004	0.459787586	1.169695841	0.710051198	0.967714316
CD274	-1.499608226	-1.330024084	-1.533256324	0.124396043	-0.317946686	0.755605817	-0.249152552	0.392923145	0.577011313	0.937785631	0.739221562	1.403044359
C8orf4	-1.247986942	-1.614128555	-0.974216952	0.025894051	-0.820948758	0.965321177	-0.143303719	0.009630543	0.717109106	0.962465543	0.524926324	1.59523818
CSF3	-1.30131128	-1.172789768	-1.2109888	-0.69013915	-0.797120263	0.959687796	0.117070369	0.311174354	0.582919588	1.047360854	0.545787029	1.60834927
IL23A	-0.896238289	-1.063311293	-0.859804363	-0.751487423	-0.972504497	0.796516517	-0.100365368	-0.249897749	0.56100677	1.219395381	0.257172923	2.059517391
C11orf96	-1.441538082	-1.194058698	-1.21104935	0.035033623	-0.194400103	1.285752709	-0.084449001	-0.505183352	0.156713457	0.841580441	0.576804189	1.734794166
LONRF1	-1.292944144	-1.321665077	-1.176525817	0.337506209	0.298406693	1.251596137	-0.341606911	-0.776576557	-0.060820238	0.78211938	0.677770322	1.622740003
FDPSP3	-0.916123915	-0.916796687	-0.915258434	-0.374655366	-0.915211283	1.521386887	-0.6130492	-0.014534921	0.131346001	0.368564253	0.559104466	2.085228199
STK40	-1.672684622	-1.385468881	-1.194984958	0.113787824	-0.414974159	0.398679333	0.325517789	0.293971335	0.102838551	1.543480207	1.209007523	0.68083006
DNAJB4	-1.613938703	-1.259995139	-0.942891339	0.269166952	0.210576842	0.145824209	-0.280844609	-0.38467732	0.020857236	1.781157023	1.394585969	0.660178879
NFKBIA	-1.442817166	-1.392773894	-1.026023491	-0.175826953	-0.169162076	0.403873313	-0.344967665	0.058107179	0.143857056	1.597401678	1.528420848	0.819911171
BAMBI	-1.436863667	-1.083913968	-1.121510993	-0.011589151	-0.132325157	-0.528245051	-0.259908324	0.426771231	0.317956885	1.698128023	1.657362897	0.474137275
RP11-356I2.4	-1.285433422	-1.266493701	-1.283209278	-0.704490016	-0.36939261	0.412569511	0.146672285	0.152805989	0.362467213	1.484273182	1.451659796	0.898571052
MIR155HG	-1.256219476	-0.881130553	-1.126596127	-0.94035376	-0.720860291	-0.046273961	-0.038829943	0.735746472	0.439885285	1.196531478	1.720579589	0.917521286
GBP5	-0.236445557	-1.506990529	-1.299318529	-0.190363542	-0.889173469	-0.530680203	0.520528663	-0.191830858	0.396218503	1.317517608	1.671540832	0.938997081
RP11-317P15.4	-0.866019676	-1.252730287	-0.699861515	-0.701926874	-0.286661878	-0.70160934	0.228441462	-0.417061705	0.230850892	1.150960688	1.764925477	1.550692757
AC116366.5	-1.096973034	-0.891713606	-1.094166778	-0.703935779	-0.308427756	0.121451313	-0.535663028	-0.245149491	0.471488173	1.282899289	1.991600916	1.00858978
AP000223.42	-1.303367037	-1.117370031	-0.826590831	-0.870367295	-0.515181266	0.449609794	-0.516704848	0.252148888	0.880304486	0.461034593	1.577679398	1.528804148
RP11-981P6.1	-1.166420105	-1.076684501	-0.865512862	-0.867463913	-0.284445012	0.910069631	-0.799357207	-0.038734973	0.697143665	0.404264548	1.458887502	1.628253228
USP54	-1.064171736	-1.010756107	-1.170020289	-0.907075783	-0.371522752	0.64031803	-0.506112791	0.235352514	0.704653979	0.243993914	1.827863283	1.377477737
CD40	-0.580384263	-0.753326705	-1.240053491	-0.842109224	-0.89576072	-0.352805705	0.0041108	-0.272866098	0.867721562	0.742756123	1.767628672	1.555089049
RN7SL751P	-1.281877406	-0.919954805	-0.916622144	-0.373216013	-0.370741597	0.276835388	-0.763093521	0.196996427	0.353499306	0.278188248	2.146178933	1.373807182
IL7	-0.470476256	-1.32567653	-1.213914207	-0.579541983	-0.601875414	-0.458372359	0.123371557	0.289899081	0.592933772	0.674528437	2.298732857	0.670391044
RP6-99M1.3	-1.994486062	-1.152161124	-1.100068192	0.337365711	0.676866086	0.783351843	-0.385371767	-0.122431287	0.185860991	0.358352545	1.382035091	1.030686165
C2CD4A	-1.117269759	-0.760522953	-1.116356335	-0.759515073	0.160375177	1.142416376	-0.865357034	-0.11569306	0.307391751	-0.062332113	1.392542847	1.794320177
MIR222HG	-1.789787681	-1.197450536	-0.987548118	0.300138766	0.338230351	0.99401122	-0.733224419	-0.313527662	0.508257476	0.393504961	1.081954767	1.405440875
ROR1-AS1	-1.289925593	-0.670084566	-0.821619716	-1.068104696	-0.821500524	0.220577646	0.018247214	-0.040314189	0.291138074	0.861318213	1.719191455	1.60107668
EIF1B	-1.180499502	-1.007302577	-1.366921261	-0.779952715	-0.73817696	0.170578222	0.248939653	0.550215776	0.317485927	0.823856209	1.553872961	1.407904266
AKAP2	-1.739915408	-0.669847617	-1.265133405	-0.443599797	0.292981841	0.732086535	-0.528669376	-0.189981803	0.093854511	0.902931575	1.441191244	1.3741017
RP11-248J18.2	-1.122712764	-1.379943968	-0.890592208	-0.689835104	0.432002176	1.053275532	-0.606185259	-0.128377874	-0.219210019	0.638366881	1.762643819	1.150568787
GORAB	-1.565510475	-1.364814232	-0.665543777	-0.076437682	0.416282217	0.670517433	-0.822637145	-0.031996063	-0.082087694	0.878970713	1.89795152	0.745305185
KRT8P9	-1.070308488	-1.229028074	-0.880070249	-0.197903961	-0.174194182	1.077242516	-0.603964049	-0.77783221	-0.091858237	0.984322726	1.502714418	1.460879789
FGF5	-1.496455067	-0.462844741	-1.850012061	-0.279905202	-0.036030664	-0.124926199	-0.049615079	0.847332583	-0.116196639	1.140413946	1.36569175	1.062547373
AMPD3	-0.707534678	-0.86734191	-1.67458837	-0.542827211	-0.79395431	-0.319537199	0.446572056	0.49604686	-0.067725465	1.261015077	1.525291782	1.244583369
IL1A	-1.437089947	-0.749774769	-1.706497176	-0.388971923	0.295966198	0.017576412	-0.319027199	0.914943663	-0.091471542	1.197446308	1.501356823	0.765543152
RP11-275I14.4	-1.163456101	-1.390276738	-1.386427325	-0.445571786	0.699012788	0.943222107	-0.444871388	-0.035377588	-0.15828573	0.78330579	1.295762083	1.302963887
RP11-352D3.2	-1.067507916	-0.88026377	-0.521175426	-0.650303072	-1.175548893	1.207328354	-0.308621561	-0.310110153	-0.172796598	0.928502051	1.235059868	1.715437118
DUSP8	-1.762770593	-1.112368968	-1.444463607	-0.179355518	0.228055262	0.747805547	0.030386256	0.444212275	-0.255571903	0.989751649	1.111342444	1.202977157
CCNL1	-1.307909416	-1.382085899	-1.202141225	0.663188545	0.403990365	0.903971211	-0.471196231	-0.619931053	-0.339163819	1.027032563	1.297494371	1.026750586
C3orf52	-1.804835287	-0.791323262	-1.53318929	0.02759014	0.820731465	0.670043883	-0.24483318	0.37496953	-0.491636828	0.929669273	1.374469625	0.66834393
ERRFI1	-1.570348754	-1.198339944	-1.051992648	0.703483874	1.031480407	0.848993277	-0.689781094	-0.509336418	-0.428647334	0.783790273	1.123194086	0.957504275
PTGS2	-1.709359312	-0.90450388	-1.568771286	0.299939963	1.0242346	0.696566117	-0.374462116	0.3301028	-0.517231206	0.568519332	1.157564145	0.997400843
GCSAM	-1.92541602	-0.82242396	-1.120117216	-0.148998107	0.567908484	1.199249512	-0.567197726	0.323260771	-0.315512172	0.519578956	1.429262936	0.860404542
SERTAD2	-1.316670737	-0.924329237	-1.853195224	0.160395311	-0.023126567	0.561964932	0.172383262	0.290189112	-0.551794833	1.351252476	1.30793015	0.825001355
SAT1	-1.098003321	-1.064397778	-1.786900478	0.286538711	0.601451382	0.010308606	-0.104005907	0.044856986	-0.551276574	1.288509952	1.527130783	0.845787639
INHBA-AS1	-1.461160434	-1.062177635	-1.113900768	1.014294815	0.521595295	0.325733902	-0.447659663	-0.457928968	-0.716081497	1.370014214	1.355931032	0.67133971
ETS2	-1.641748803	-0.749386021	-1.460958643	-0.014681079	0.431211748	0.340881327	-0.069495944	0.358939421	-0.714596806	1.512446018	1.427786663	0.57960212
ARNTL2-AS1	-1.077249848	-1.176418606	-1.278677017	-0.075495405	0.570542166	0.695354425	-0.820943861	0.197641215	-0.743816106	0.94028252	1.490088308	1.278692207
MIR29A	-1.389849839	-1.087168823	-1.021330457	0.66418526	0.789106353	1.040215452	-0.750425459	-0.618719358	-0.685582251	0.883650817	1.346089302	0.829829004
USP53	-1.299111992	-0.926140475	-1.580120712	0.651050705	0.619912856	0.556124549	-0.424738065	0.063570261	-0.858099918	1.040298757	1.583841746	0.573412288
GEM	-0.883468797	-1.187075348	-1.490663446	0.987511551	0.011882378	0.815238321	-0.095564829	-0.959238883	-0.419388511	1.350364444	0.56745363	1.30294949
DUSP5	-1.355684835	-0.746189324	-1.86173785	1.276440114	0.691210969	0.667078557	-0.02695577	-0.322467681	-0.59342795	1.249312353	0.664285277	0.358136139
KDM7A	-0.989141407	-1.329671385	-1.433776578	0.409004123	0.232875865	1.183696929	0.211922196	-0.627681018	-0.816792297	1.068459407	0.779849153	1.311255012
TRIB1	-1.468429295	-0.936353092	-1.411417971	1.206942605	0.512715283	0.6842865	-0.518737351	-0.630704403	-0.126238709	1.535255064	0.372791917	0.779889453
RND3	-1.182858269	-1.506876396	-1.469609804	0.799857913	0.406611416	0.810585167	0.024291142	-0.73695013	0.017359817	1.14044435	0.38840255	1.308742243
HSPB8	-1.347199994	-0.675245748	-1.763184022	0.147923997	0.079412149	-0.190834336	0.086387742	-0.072395232	-0.274802981	1.577124748	1.318479534	1.114334142
PPP1R15A	-0.955194356	-1.340546236	-1.066522352	0.743843737	0.125366615	0.747349317	-0.605196247	-0.913428395	-0.412382919	1.540477059	1.064391176	1.071842601
ATF3	-1.285231939	-1.262092611	-1.2664391	0.882909	0.39581565	0.733911357	-0.50087606	-0.763090844	-0.260967294	1.315386386	0.896546026	1.11412943
ABL2	-1.514053366	-1.061081646	-1.624685994	0.670261445	0.544394031	0.576024174	-0.328449543	-0.053750968	-0.441952072	1.284896651	1.043296939	0.905100349
PIM3	-1.299684325	-1.22927959	-1.251503093	-0.05750483	-0.675553238	0.829069939	-0.033462926	-0.10417152	-0.031012762	1.489202039	1.006268486	1.357631822
ZNF697	-1.353757232	-1.604580098	-1.242441033	0.903003453	0.40868835	0.286306583	-0.097256335	-0.403788096	-0.11893012	1.502976263	1.033364209	0.686414055
C9orf47	0.034028053	-0.528369635	0.159363394	-0.915088669	-1.541297131	-1.200173286	1.623885277	0.840894868	1.565498674	0.061517744	-0.394489071	0.294229782
APOL2	0.179498143	-1.088250539	-0.256495313	-0.031816972	-1.537514969	-0.891283937	1.858483079	0.706908814	1.489397884	0.209891237	-0.582340276	-0.05647715
ENC1	-0.35402626	-0.188127066	-0.427839694	-1.104759684	-1.339906296	-0.757129756	1.51768919	1.558051619	1.399778996	0.3742095	-0.279735581	-0.398204969
RBM47	0.481792488	-1.197619452	-0.85578321	-0.029186073	-1.279285002	-0.570524402	1.752413561	0.165572906	1.441593835	0.569960991	-0.95437797	0.475442327
CD34	0.092373052	-0.925375432	0.370283023	-0.276009525	-1.113084473	-0.234747884	1.246895775	0.090186274	2.104470129	0.503912018	-1.501477945	-0.357425011
CDC42EP2	-1.357315485	-0.915264996	0.011802613	-0.132013235	-1.19953151	0.57169663	0.440508993	0.794051616	2.152414799	0.226477271	-0.949190369	0.356363673
RP11-693N9.2	0.111448914	-0.598307926	-0.217970209	-0.597042361	0.409900508	-0.596799561	1.710475925	2.029681501	0.409972284	-0.596040693	-1.033482079	-1.031836304
CBR3	-0.021454613	-0.956462938	0.274457689	-0.886897286	-1.318075138	0.006613961	1.934125939	0.629136549	1.258270656	-0.506813931	-1.050083598	0.637182709
FAM101A	-1.3375858	-0.394569364	0.243304598	-1.269353644	-0.747336821	-0.039790567	0.580446111	1.315512123	1.764421587	-0.270340529	-0.805193756	0.960486062
TMEM217	-0.975912216	0.424103236	-0.060050344	-0.646429299	-0.767630275	-1.366662726	0.798014764	1.382751213	2.0172583	-0.108447913	-0.589754857	-0.107239885
FZD7	-0.103589478	-0.57872536	0.253332828	-0.978200961	-1.42729328	-0.528376977	1.661164877	1.213331642	1.623791684	-0.290912888	-0.361588734	-0.482933353
AXIN2	0.335533798	-0.631574223	0.268061893	-0.40811066	-1.163128379	-1.196969231	0.716560855	0.46680457	2.337553691	-0.212762368	-0.995129396	0.483159451
CXCL6	-0.83093321	-0.570965497	-0.039335187	-1.145778279	-1.063899398	0.53105543	0.591650867	0.709606574	2.149306966	-0.431599405	-0.841409591	0.942300729
ELF3	-0.781194679	-0.63629116	-0.510197125	-1.062170591	-0.863886069	0.177786459	1.08889367	0.585162291	2.121183247	-0.70431606	-0.457064848	1.042094866
CCL1	-0.785339862	-0.609021173	-0.194078284	-0.785328898	-0.784482236	-0.195245051	0.106383283	1.588089313	2.286337816	-0.784819966	-0.31833793	0.475842988
RP4-607I7.1	-0.627844891	-0.841983562	-0.133740441	-1.002222885	-0.999896448	-1.28699928	1.053570034	1.145161107	1.808325636	0.701435797	0.186849131	-0.002654198
RP11-75L1.2	-0.850183011	-1.034298332	-1.492364539	-0.76741588	-0.687096801	-0.416156158	1.223998877	1.066632588	1.50687653	0.251893574	0.76979857	0.428314582
TNFSF15	-0.984952293	-0.048055469	-1.528499922	-1.148643413	-0.567444163	-0.34020957	1.310261286	1.424572054	1.392634126	0.0441615	-0.048042894	0.494218759
CCL2	-1.384805195	-0.723374024	-0.01217256	-1.438967611	-1.251280378	0.272347477	0.644060174	1.226511305	1.498570068	0.175581742	0.214666755	0.778862248
GATM	-0.635767622	-0.150435178	-1.127688419	-0.975472523	-0.511610894	-0.974941371	0.777058453	2.232951267	1.174954504	-0.097666622	0.385230605	-0.096612199
AC020571.3	-0.745425932	0.624815913	0.042244576	-1.676149816	-0.148544848	-1.152576257	0.505872514	1.47904149	1.400442426	-0.743125465	0.858541505	-0.445136105
HAS3	-0.878272052	0.770547294	-0.888986693	-1.325769558	0.1489107	-0.96132451	0.217753105	1.788512328	1.49695596	-0.7483481	0.086786181	0.293235344
