# Supplementary material for: The endocannabinoid anandamide has an anti-inflammatory effect on CCL2 expression in vascular smooth muscle cells
Source: Basic Res Cardiol. 2020 Apr 22;115(3):34. doi: 10.1007/s00395-020-0793-3 (PMC7176595; doi:10.1007/s00395-020-0793-3)
Supplement: Supplementary file 2 — Supplementary file2 (PDF 311 kb) [file 395_2020_793_MOESM2_ESM.pdf]

The Endocannabinoid Anandamide has an anti-inflammatory effect on CCL2 expression in vascular smooth muscle cells

Beatrice Pflüger-Müller[1,2], James A. Oo[1,2], Jan Heering[4], Timothy Warwick[1,2], Ewgenij Proschak[6], Stefan Günther[3], Mario Looso[3], Flávia Rezende[1,2], Christian Fork[1,2], Gerd Geisslinger[4,5], Dominique Thomas[5], Robert Gurke [4,5], Dieter Steinhilber[4,6], Marcel Schulz[7], Matthias S. Leisegang[1,2] and Ralf P. Brandes[1,2*]

[1] Institute for Cardiovascular Physiology, Goethe University, 60590 Frankfurt am Main, Germany,
[2] German Center for Cardiovascular Research (DZHK), Partner site Rhein Main, Frankfurt am Main, Germany,
[3] Max-Planck-Institute for Heart- and Lung Research (MPI-HLR), 61231 Bad Nauheim, Germany,
[4] Fraunhofer Institute for Molecular Biology and Applied Ecology IME, Branch for Translational Medicine and Pharmacology TMP, 60438 Frankfurt am Main, Germany,
[5] Pharmazentrum Frankfurt/ ZAFES, Institute of Clinical Pharmacology, Faculty of Medicine Goethe-University, 60590 Frankfurt am Main, Germany,
[6] Institute of Pharmaceutical Chemistry, Goethe-University, 60438 Frankfurt am Main, Germany,
[7]Vascular Research Centre, Goethe-University, 60596 Frankfurt am Main, Germany

Short running title: Anandamide and vascular anti-inflammatory actions

Supplemental Tables

Table 1 siRNAs used in this study

Gene	Cat. No.	Company
**NCoR1**	#HSS114352	Stealth siRNA, Thermofisher
**Sin3A**	#J-012990-05-0002	Dharmacon
**LCoR1**	#J-015742-17-0002	Dharmacon
**RCOR1**	#J-014076-06-0002	Dharmacon
**SMRT**	#HSS114356	Stealth siRNA, Thermofisher

Gene	Cat. No.	Company
**HDAC4**	#HSS114673	Stealth siRNA, Thermofisher
**HDAC5**	#HSS173323	Stealth siRNA, Thermofisher
**HDAC7**	#HSS147499	Stealth siRNA, Thermofisher
**HDAC9**	#HSS114607	Stealth siRNA, Thermofisher
**siScr1**	#12935-200	Stealth siRNA, Thermofisher
**siScr2**	#12935-300	Stealth siRNA, Thermofisher
**siScr3**	#12935-400	Stealth siRNA, Thermofisher

Table 2 Primer sequences for RT-qPCR

Gene	Primer	Sequence (5‘-3‘)
**Human CCL2**	forward	CAA GCA GAA GTG GGT TCA GGA T
	reverse	TTA GCT GCA GAT TCT TGG GTT GT
**Murine CCL2**	forward	CCA CTC ACC TGC TGC TAC TCA TTC
	reverse	GTC AC TCC TAC AGA AGT GCT TGA GG
**Human NCoR1**	forward	GAT CAC CAG GTC CAT GAC AAA C
	reverse	TTC GTT CCC ACC ATT TTA GCA A
**Human GAPDH**	forward	TGC ACC ACC AAC TGC TTA GC
	reverse	GGC ATG GAC TGT GGT CAT GAG
**Murine α-Actin**	forward	ACA GAG GCA CCA CTG AAC CCT AAG
	reverse	ACA ATC TCA CGC TCG GCA GTA GTC
**human HDAC4**	forward	GAA ACG AGC TTG ATC CTC TCC CAG
	reverse	GCT TCA CGC CCA CGG ACA GCG AG

Gene	Primer	Sequence (5‘-3‘)
**human HDAC5**	forward	TCT GAG GCT TGT GTC TCG GCT CTG
	reverse	AGG CTC CTG CTC CAT GGG CTC CTC
**human HDAC7**	forward	TCT TCT GGG TAA CAG GGT GGA TC
	reverse	ACC AGC TGC TCC GAG GGC CTA TC
**human HDAC9**	forward	TCA GGA CCA TCG TGA AGC CTG TG
	reverse	TGC AAG TGG CTC CAG CTC ATT TCC

Table 3 Primer sequences for ChIP qPCR

Locus	Primers	Sequence (5’-3’)
**CCL2 TSS**	forward	TCC CTC CTC CTG CTT GAC TC
	reverse	AGT CTC AGC CTC TCG GTT CC
**CCL2 -200 bp**	forward	GCT TCA GAG AAA GCA GAA TC
	reverse	TTC ACT GCT GAG ACC AAA TG
**CCL2 -600 bp**	forward	AGC AGG CTA TTT AAC CCT TC
	reverse	GGC ATA GAC AGC ATG TAG TG
**CCL2 -1000 bp**	forward	ATA TTC CTG GCA GAG TAA GC
	reverse	TCA TAG AAG CCT AGC AGA AC
**GAPDH**	forward	TGG TGT CAG GTT ATG CTG GGC CAG
	reverse	GTG GGA TGG GAG GGT GCT GAA CAC

Table 4 Primary antibodies used in this study

Protein	Order No.	species	company
**MCP-1**	# sc-52877	rabbit	Santa Cruz
**β-Tubulin**	# sc-9104	rabbit	Santa Cruz
**Topoisomerase I**	# sc-5342	goat	Santa Cruz

Protein	Order No.	species	company
**HDAC4**	# A303-467	rabbit	Bethyl Laboratories
**NCoR1**	#LS-C108878-100	mouse	LsBio (für PLA)
**NCoR1**	# A301-145	rabbit	Bethyl Laboratories
**p65 NFkB**	# sc-109	rabbit	Santa Cruz
**H3K4me1**	# C15410037	rabbit	Diagenode
**H3K4me3**	# pAb-003-050	rabbit	Diagenode
**H3K27ac**	# pAB-174-050	rabbit	Diagenode
**H3K27me3**	# pAB-069-050	rabbit	Diagenode
**H3-pan**	# C15200011	mouse	Diagenode
**Polymerase II**	# C15100055	mouse	Diagenode
**β-Actin**	# A1978	mouse	Sigma
**p-JNK**	# 4688	rabbit	Cell Signaling
**JNK**	# sc-1648	mouse	Santa Cruz
**p38**	# ab31828	mouse	Abcam
**p-p38**	# 9211	rabbit	Cell Signaling
**ERK1/2**	# 4696	mouse	Cell Signaling
**p-ERK1/2**	# 9101	rabbit	Cell Signaling
**IgG**	# C15410206	rabbit	Diagenode
